# Targeting Cell Death Mechanism Specifically in Triple Negative Breast Cancer Cell Lines

**DOI:** 10.3390/ijms23094784

**Published:** 2022-04-26

**Authors:** Lavinia-Lorena Pruteanu, Cornelia Braicu, Dezső Módos, Maria-Ancuţa Jurj, Lajos-Zsolt Raduly, Oana Zănoagă, Lorand Magdo, Roxana Cojocneanu, Sergiu Paşca, Cristian Moldovan, Alin Iulian Moldovan, Adrian Bogdan Ţigu, Eugen Gurzău, Lorentz Jäntschi, Andreas Bender, Ioana Berindan-Neagoe

**Affiliations:** 1 Department of Chemistry, Centre for Molecular Science Informatics, University of Cambridge, Cambridge CB2 1EW, UK; pruteanulavinia@gmail.com (L.-L.P.); dr.dezso.modos@gmail.com (D.M.); ab454@cam.ac.uk (A.B.); 2 MedFuture Research Center for Advanced Medicine, “Iuliu Hațieganu” University of Medicine and Pharmacy, 400377 Cluj-Napoca, Romania; moldovan.cristian1994@gmail.com (C.M.); alin.moldovan92@yahoo.ro (A.I.M.); adrianbogdantigu@gmail.com (A.B.Ț.); 3 Department of Chemistry and Biology, North University Center at Baia Mare, Technical University of Cluj-Napoca, 4800 Baia Mare, Romania; 4 Research Center for Functional Genomics, Biomedicine and Translational Medicine, “Iuliu Hațieganu” University of Medicine and Pharmacy, 400337 Cluj-Napoca, Romania; ancajurj15@gmail.com (M.-A.J.); raduly.lajos78@gmail.com (L.-Z.R.); zanoaga.oana@gmail.com (O.Z.); lorand.magdo@gmail.com (L.M.); cojocneanur@gmail.com (R.C.); pasca.sergiu123@gmail.com (S.P.); ioana.neagoe@umfcluj.ro (I.B.-N.); 5 Department of Pharmaceutical Physics-Biophysics, “Iuliu Hațieganu” University of Medicine and Pharmacy, 400349 Cluj-Napoca, Romania; 6 Environmental Health Center, 400240 Cluj-Napoca, Romania; egurzau@ehc.ro; 7 Institute for Doctoral Studies, Babeş-Bolyai University, 400084 Cluj-Napoca, Romania; lorentz.jantschi@chem.utcluj.ro; 8 Department of Physics and Chemistry, Technical University of Cluj-Napoca, 400641 Cluj-Napoca, Romania

**Keywords:** triple negative breast cancer, breast cancer, gene expression, arsenate, microarray, mode of action

## Abstract

Triple negative breast cancer (TNBC) is currently associated with a lack of treatment options. Arsenic derivatives have shown antitumoral activity both in vitro and in vivo; however, their mode of action is not completely understood. In this work we evaluate the response to arsenate of the double positive MCF-7 breast cancer cell line as well as of two different TNBC cell lines, Hs578T and MDA-MB-231. Multimodal experiments were conducted to this end, using functional assays and microarrays. Arsenate was found to induce cytoskeletal alteration, autophagy and apoptosis in TNBC cells, and moderate effects in MCF-7 cells. Gene expression analysis showed that the TNBC cell lines’ response to arsenate was more prominent in the G2M checkpoint, autophagy and apoptosis compared to the Human Mammary Epithelial Cells (HMEC) and MCF-7 cell lines. We confirmed the downregulation of anti-apoptotic genes (MCL1, BCL2, TGFβ1 and CCND1) by qRT-PCR, and on the protein level, for TGFβ2, by ELISA. Insight into the mode of action of arsenate in TNBC cell lines it is provided, and we concluded that TNBC and non-TNBC cell lines reacted differently to arsenate treatment in this particular experimental setup. We suggest the future research of arsenate as a treatment strategy against TNBC.

## 1. Introduction

Breast cancer is a common female malignancy, representing 11.6% of all cancer cases worldwide, Ref. [1] particularly in developing countries [2,3,4]. Among all breast cancer subtypes, triple negative breast cancer (TNBC) is differentiated from other types of breast cancer by not expressing three receptors, namely the Estrogen receptor, Progesterone receptor and the receptor tyrosine kinase Her2/neu [3,4,5]. TNBC occurs more often in younger women and it is more difficult to identify on a mammogram [2,3,4]. Furthermore, TNBC tends to grow faster than other types of cancer and it frequently recurs [3,6].

Standard therapy across breast cancer subtypes includes surgery, chemotherapy, and radiation. Non-selective chemotherapies are especially important for TNBC patients, because no targeted therapy is available. Also, for those patients who have developed metastases, surgery alone is not sufficient, and additional general chemotherapy is necessary [4,6,7,8,9,10,11]. Hence, treatment options for TNBC differ from other types of breast cancer, and they are generally more limited [3,5]. All of these factors necessitate potential novel chemotherapeutic agents, ideally with at least limited selectivity.

Among non-specific cancer treatments, arsenic-derived compounds are commonly used [12,13,14]. In the human body, inorganic arsenic compounds are converted to arsenite (arsenic in the +3 oxidation state) and arsenate (arsenic in the +5 oxidation state). Arsenite is significantly more toxic than arsenate, which is related to both reactivity and transport [12,13,14]. Upon entering the cell, arsenate can be reduced to arsenite, leading to both chemical species being present depending on the redox state of the cell [15,16,17].

Arsenate is known to cause direct and indirect DNA damage through reactive oxygen species, [18] and can affect the DNA repair mechanism at low concentrations [19]. Increased inorganic phosphate transport observed in the MDA-MB-231 cell line may be associated with the higher energy demands linked to its metastatic phenotype and can increase the arsenate intake as well [20]. Arsenic has been studied more extensively on the transcriptomic level due to its clinical relevance, [21] however the same is not yet true for arsenate, which is the contribution of the current work [20]. On the mechanistic level, the activity of arsenate is largely unknown in breast cancer. Here we attempted to unravel the effects of arsenate on TNBC cells in comparison with one double positive breast cancer (DPBC) and one normal breast cell line. In particular, we used the normal breast cell line HMEC (Human Mammary Epithelial Cells), the DPBC cell line MCF-7, as well as the TNBC cell lines Hs578T and MDA-MB-231. HMEC and MCF-7 possess intact DNA repair mechanisms according to the COSMIC cell line encyclopedia, while the two TNBC cell lines are p53 mutants [22].

The purpose of this study was to investigate whether arsenate can be a selective chemotherapeutic agent against TNBC cell lines. For this, we examined the response to arsenate treatment in autophagy, apoptosis and cell proliferation via phenotypic and transcriptomics readouts. Firstly, we evaluated the effect of arsenate treatment on the cellular level. We performed functional tests, including colony assay formation, autophagy and apoptosis investigation using fluorescence microscopy, and measured cytoskeletal response by immunofluorescence staining followed by confocal microscopy evaluation and dark field microscopy for morphologic alteration. Then, after seeing that arsenate causes apoptosis specifically in the Hs578T and MDA-MB-231 cell lines, we investigated whether there is any mechanistic change in the transcriptome of the cell lines responding to the low arsenate concentration using cDNA microarrays and regularized discriminant analysis (RDA). Finally, findings from the gene expression analysis were validated by qRT-PCR, and at the protein level by ELISA and immunofluorescence confocal microscopy.

## 2. Results

### 2.1. Arsenate Exposure Decreases Colony Formation in Cancer Cell Lines

**Figure 1:**
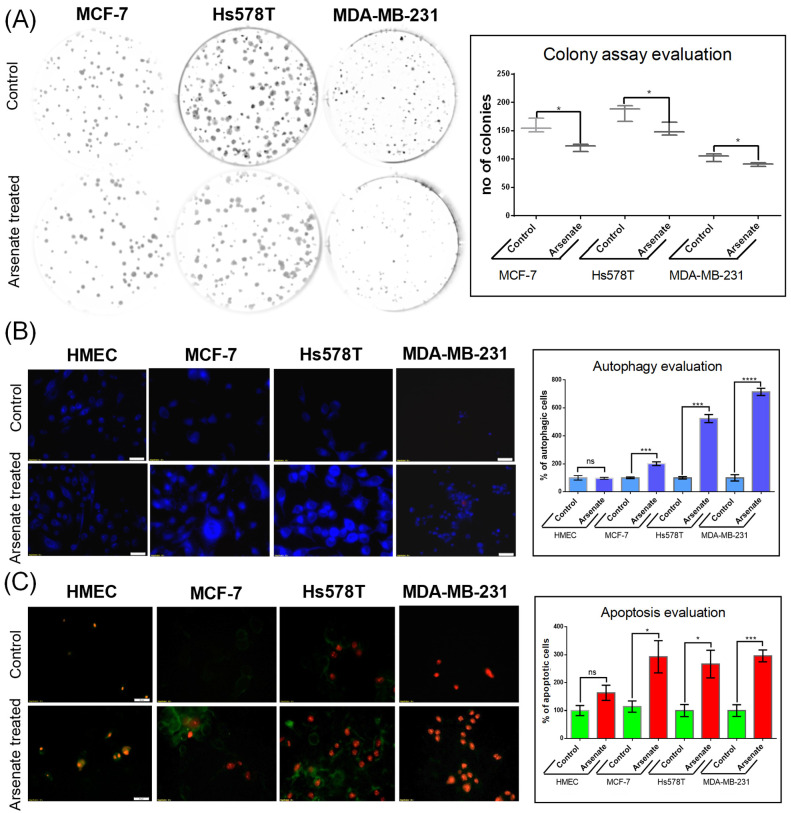
Evaluation of cellular effect of arsenate. (**A**) Arsenate treatment (50 nM) affects the ability of breast cancer cell lines to form colonies at 24 h after arsenate treatment. On the left side of the figure are representative images of the colony formation assays for each cell line, while on the right side boxplots for the number of colonies are shown. (* *p* < 0.05 two sided *t*-test treated vs. untreated cells *n* = 3). Arsenate significantly decreased the colony forming capacity of all cancer cell lines used in this study (while the normal HMEC cell line used as a control is not able to form colonies in any case). (**B**) Autophagy evaluation by fluorescence microscopy following exposure to 50 nM arsenate on different cell lines (20× magnification). This experiment emphasizes an increase of MDC-labeled vesicles as an effect of arsenate exposure in tumor cells, which is less profound in normal cells 24 h after treatment. Compared to control, the number of autophagic cells was significantly increased 24 h after arsenate treatment. (*** *p* < 0.001, **** *p* < 0.0001 two sided *t*-test, treated vs. untreated cells *n* = 3) (**C**) Apoptosis evaluation in the same experimental conditions at 24 h after arsenate treatment. Green fluorescence is Annexin V-FITC and red fluorescence is propidium iodide (PI). Annexin V-FITC around the membrane displays apoptotic cells, only red fluorescence in the nucleus displays the necrotic cells and double staining is specific for late apoptotic cells. Large numbers of fluorescent Annexin V-FITC and Annexin V-FITC/PI was observed in tumoral cells treated with arsenate; in contrast, few fluorescent cells were observed in the HMEC control cells. The number of cells were counted with the apoptotic markers and normalized with the untreated controls. (* *p* < 0.05, *** *p* < 0.001 two sided *t*-test, treated vs. untreated cells *n* = 3).

We first assessed the effect of arsenate exposure on colony formation capacity, which is a widely used test to assess the sensitivity of tumor cell lines to therapeutic agents [23]. The test evaluates the clonogenic capacity, responding to much lower doses than the standard MTT test. The result (Figure 1A) shows that arsenate concentration of 50 nM (final concentration in the cell culture medium) has the capacity to attenuate the colony formation rate in all three breast cancer cell lines (for MCF-7, 0.76 fold; Hs578T 0.83 fold; MDA-MB-231 to 0.87 fold of control). The effect of arsenate treatment on the ability of forming colonies has been shown in previous studies, Ref. [24] and the current results are in agreement with that. (The HMEC cell line was unable to form colonies, since it is a non-tumorous cell line).

### 2.2. Effect of Arsenate on the Modulation of Apoptosis and Autophagy in MCF-7, Hs578T, and MDA-MB-231 Cells

Given the decrease of colony formation ability described above, we next investigated its mechanistic background, focusing on apoptosis and autophagy. We stained the cells with monodansylcadaverine (MDC), a fluorescent marker for the acidic endosomes, lysosomes, and late-stage autophagosomes [25]. An increase of the MDC signal was observed in the case of treated cancer cell lines compared to untreated cells (Figure 1B), which generally labels later stages of the degradation processes, as opposed to being a specific marker for autophagy [25]. Arsenate treatment increased the number of autophagy vacuoles containing MCF-7 cells by a factor of 1.91 compared to control (*p* = 0.0004). However, the increase is not observed for arsenate treated vs. untreated HMEC cells (Figure 1B; *p* > 0.05 in a *t*-test). The number of autophagic cells in the Hs578T and MDA-MB-231 cell lines increased significantly (by a factor of 5.31 and 6.43, *p* = < 0.0001 and 0.0001 in a *t*-test, respectively).

The cells were then simultaneously stained with Annexin-V and FITC/PI to visualize apoptosis. Only Annexin-V FITC staining visualizes early apoptosis, meanwhile both Annexin-V and FITC PI are markers for late apoptosis. An increased apoptotic rate was observed in the triple negative breast cancer cell lines as follows. In the Hs578T cell line, both the early and late apoptotic state was visible, while in case of the MDA-MB-231 cells, the majority of cells appeared in the late apoptotic phase (70–90%; Figure 1C). The number of apoptotic cells also increased in the DPBC MCF-7 cell line (by a factor of 2.82; *p* = 0.026 in a *t*-test), but not in the HMEC cell line (*p* > 0.05).

These data suggest the involvement of apoptosis and likely autophagy as a response to arsenate exposure, which, based on the data shown here, is more pronounced in the TNBC cancer cell lines. Consistent with that, arsenic in oxidation state +3 has previously been found to interfere with intrinsic and extrinsic apoptosis and autophagy mechanisms in the breast cancer cell lines MCF-7 and MDA-MB-231 [26]. Arsenic trioxide was found to suppress cell growth, to stimulate apoptosis, and to be involved in retarded cell invasion by interfering with coding and non-coding gene regulation [27,28,29,30].

Autophagy has been presented in previous literature as a protective mechanism against arsenite induced oxidative stress, which causes genome damage [31]. Meanwhile, there is no literature information related to the modulation of autophagy by arsenate. Based on the current study, we can conclude that arsenate exposure likely activates autophagy (with some ambiguity from the MDC marker used as in our previous studies [32,33]) and (more certainly) apoptosis, and hence similar cellular mechanisms as arsenite. In our study the activation of autophagy observed by fluorescence microscopy is supported by microarray data. The idea of this study was to demonstrate how complex the mechanistic effects of arsenate are. On this occasion we did not focus on a single mechanism like the autophagy. Microarray data shows that arsenate’s effects are complex, emphasizing the crosstalk among the different signalling pathways. This observation was is confirmed as well in publications such as [34].

### 2.3. Dark-Field Microscopic and Cytoskeletal Evaluation

**Figure 2:**
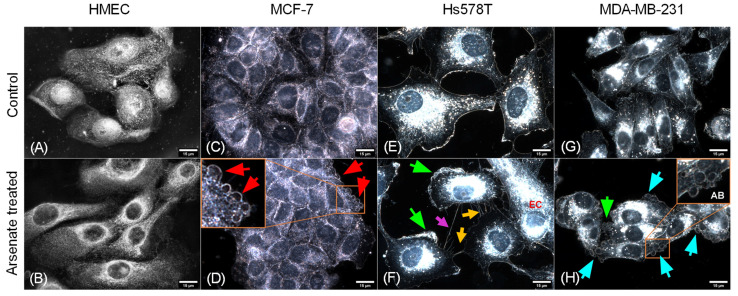
Effect of arsenate treatment on four different cell lines visualized using dark field microscopy. Magnification of 15 μm. (**A**) HMEC untreated cells, (**B**) HMEC arsenate treated cells, (**C**) MCF-7 untreated cells, (**D**) MCF-7 arsenate treated cells, (**E**) Hs578T untreated cells, (**F**) Hs578T arsenate treated cells, (**G**) MDA-MD-231 untreated cells, (**H**) MDA-MD-231 arsenate treated cells. Red arrows (**D**) indicate apoptotic bodies; green arrows (**F**,**H**) indicate thickened and fragmented cell membranes. EC (orange—(**F**)) elongated cell, orange arrows (**F**) indicate apoptopodia-like projections, magenta arrows (**F**) indicate tunneling nanotubes, cyan arrows (**H**) show apoptotic bodies labeled AB. No important alterations can be seen for normal cell lines; meanwhile, in the case of tumor cells, the activation of apoptotic mechanisms can be observed (indicated by red arrows), which is more pronounced for TNBC cells.

We next used dark field microscopy to assess cellular morphology, which allows for the assessment of cytoskeleton alterations and hence the actin and tubulin status of a cell. It can also resolve more discrete features such as membrane disorganization, blebbing and apoptotic bodies. In the case of HMEC cells, only a slight modification of morphology was observed (Figure 2). The cells appear slightly elongated in the arsenate treated group compared to the control. Nuclei have a normal round/elliptical shape with no signs of fragmentation. After arsenate exposure the cell membrane of TNBC cells became thick and fragmented (Figure 2F,H, green arrows).

Cellular stress becomes visible through abnormal elongated cells for MCF-7 and Hs578T cells and irregular nuclei surrounded by sparse apoptotic bodies (Figure 2D,F). Numerous apoptotic bodies can be seen in the case of arsenate treated cells (Figure 2H), and the apical membrane (Figure 2F) shows signs of breakage and a higher degree of disorganization. Apoptopodia-like projections are prominent in the case of Hs578T cells (Figure 2F), which are less pronounced for MDA-MB-231 and MCF-7 and not present in the case of HMEC cells. In the case of Hs578T, the presence of cells with abnormally high nuclear displacement and the formation of tunneling nanotubes (Figure 2F) can be observed. All of this shows that HMEC cells are not going through the same strong apoptotic response that is observed for the tumor cells.

**Figure 3:**
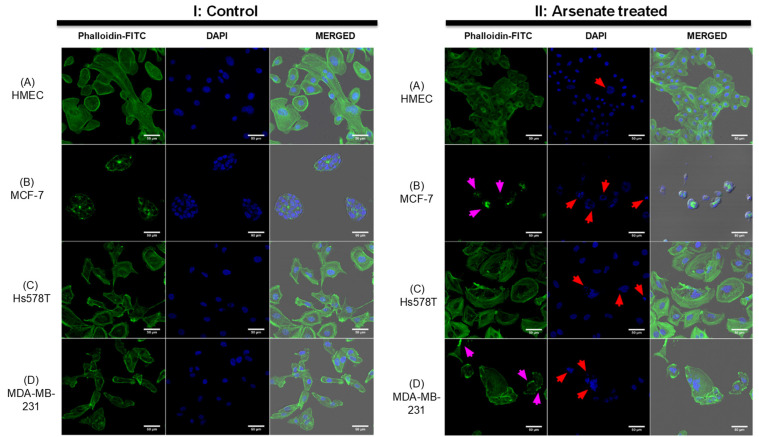
Changes in the cytoskeleton after arsenate exposure (**II**) with cytoskeleton staining by Phalloidin-FITC and nucleus staining by DAPI in comparison to control (**I**). Magnification of 50 μm. (**A**)—HMEC; (**B**)—MCF-7; (**C**)—Hs578T; (**D**)—MDA-MB-231. Note in (**II**) the completely disorganized actin cytoskeleton in the case of the MCF-7 cell line and the increased amount of actin filaments at the cell membrane of the MDA-MB-231 cell line affecting cytoskeletal organization. Red arrows point to irregular or fragmented nuclei and magenta arrows indicate cytoskeleton damage. Alterations are more prominent in all cancer cell lines compared to the HMEC cell line where the actin filaments are not affected and nuclear damage is insignificant.

To assess whether the morphological response of the cellular cytoskeleton to arsenate treatment is in accordance with the dark field microscopy images, we next stained the actin cytoskeleton with Phalloidin-FITC dye and the cell nuclei with DAPI staining, visualized in Figure 3. The response of HMEC cells (Figure 3AI,II) to arsenate exposure was reduced compared to the cancer cell lines, and it can be observed that the cells are now more compact and have a slightly elongated shape. Also, the nucleus and cytoplasm area of HMEC cells is reduced as a result of arsenate exposure. Some larger nuclei are still visible in the case of HMEC cells, which indicates a stress response; however, the nuclei are not fragmented. In contrast, in the case of breast cancer cells, the nuclear fragmentation is more pronounced in TNBC cells (Figure 3BII).

Alteration of the cytoskeletal organization is overall more pronounced in breast cancer cells. Hs578T cells treated with arsenate do not present significant alterations to the cytoskeleton (Figure 3CII); however, irregular and fragmented nuclei are now present as an effect of arsenate treatment (indicated by red arrows). In the case of MDA-MB-231 untreated cells (Figure 3DI), we can observe normal morphology; meanwhile, those cells treated with arsenate (Figure 3DII) have giant multinucleated cells and the cytoskeleton staining is now stronger on the edges of the membrane. This is in agreement with previous work in that arsenic trioxide is a chemical agent recognized to produce cytoskeletal injury [35]. It has also been previously demonstrated in a separate study that arsenic trioxide affects the cytoskeleton, cell adhesion and epithelial mesenchymal transition- related genes [36]. What is novel in this work though is that arsenate’s therapeutic stress produces pro-apoptosis signals largely selectively in TNBC cells (based on dark-field microscopic and cytoskeletal evaluation). We then wanted to understand these changes at the transcriptomic level.

### 2.4. Mode-of-Action Analysis of Arsenate Treatment Based on Gene Expression Data

**Figure 4:**
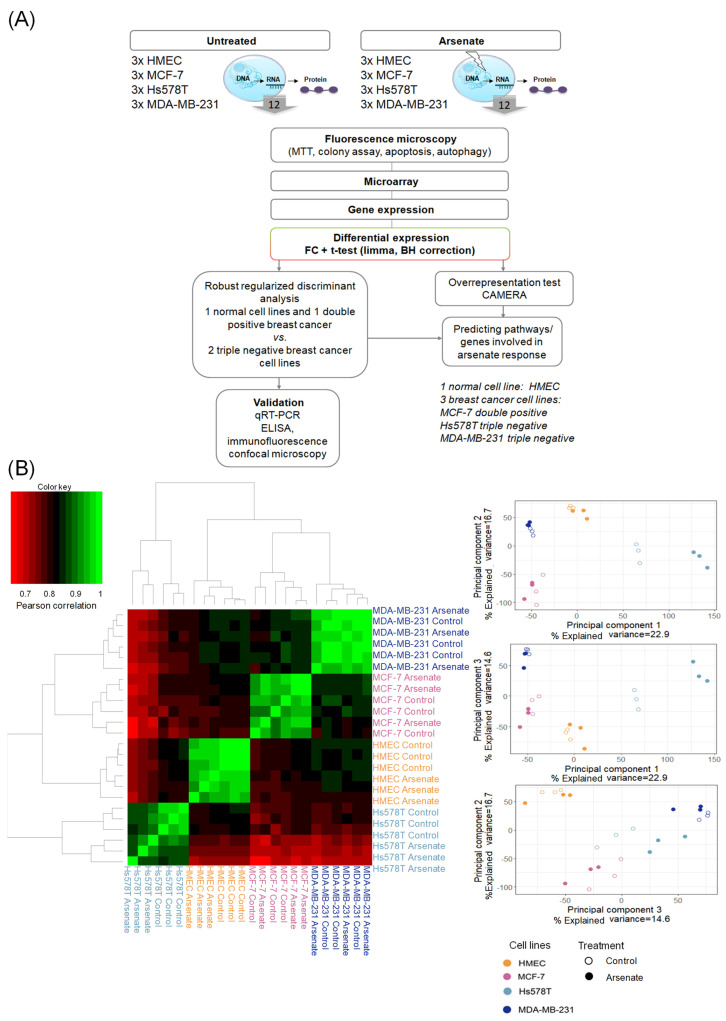
(**A**) Workflow of this study. First, we started with phenotypic readouts of different methods from the control (HMEC) and the three different breast cancer cell lines (MCF-7, Hs578T and MDA-MB-231) with and without arsenate treatment. Next, microarray data were collected from three replicates each of the four cell lines in each condition. Subsequently, we determined differentially expressed genes using fold change (FC) and the False Discovery Rate (FDR)-corrected *t*-test, CAMERA. The response to arsenate between the two TNBC and the DPBC and normal cell lines was compared by using Robust Regularized Discriminant Analysis. From the resulting centroid of data, GSEA (gene set enrichment analysis) was performed in order to identify the involved Gene Ontology Biological Processes (GO-BP) and pathways. Representative genes were selected to validate the differentially expressed genes by qRT-PCR, ELISA, and fluorescence microscopy. (**B**) Similarity of cell lines and treatment conditions based on Pearson correlation and principal component analysis (PCA). It can be seen that the cell type causes bigger differences in gene expression space than treatment conditions (panel **A**). In (panel **B**) the arsenate’s response has no specific direction compared to untreated samples; however, treated and untreated samples are generally distinguishable.

We next investigated the mode-of-action of arsenate treatment in the four different cell lines based on gene expression data (see Figure 4A for the experimental workflow and methods section for experimental details). A Pearson correlation matrix analysis visualized as a heatmap (shown in Figure 4B) showed that the first differentiating factor between samples is the cell line, and only the second one is the arsenate treatment. This is in agreement with previous experiments in breast cancer cell lines and their response to chemotherapeutics [37]. We next used principal component analysis (PCA) to visualize differences between the different cell lines and treatment conditions further, the results of which are shown in Figure 4B. It can be seen, in agreement with the correlation analysis, that the four cell lines are located in rather distinct locations of PCA space. Arsenate treated cells, as a whole, are not distinct from untreated cells in a specific direction in the first three principal components; however, they generally differ from the non-treated cell lines (Figure 4B).

We next evaluated gene expression on an individual gene and pathway level. First, we found the number of differentially expressed genes in the four arsenate-treated cell lines, which were 81 for HMEC, zero for MCF-7, 1231 for Hs578T and 275 for the MDA-MB-231 cell line (with a *q*-value < 0.1 in a Benjamini-Hochberg False Discovery Rate-corrected *t*-test and |log2 FC| > 1; see Table S3 for details in the Supplementary Materials). This seems to an extent surprising given the toxicity of arsenic [38], and one reason might be that the concentration of arsenate (50 nM) is relatively low. Furthermore, the two TNBC cell lines had a stronger response than the two other cell lines investigated here. However, these differentially expressed genes were not enriched in any gene ontology biological process, (FDR > 0.1) nor were they enriched by using the CAMERA method for gene-set enrichment analysis (FDR > 0.1). To distinguish the weak transcriptomics signal, we compared the response of the TNBC and DPBC and normal cell lines.

### 2.5. Arsenate Response in Triple Negative Cell Lines vs. Double Positive and Normal Cell Line

**Figure 5:**
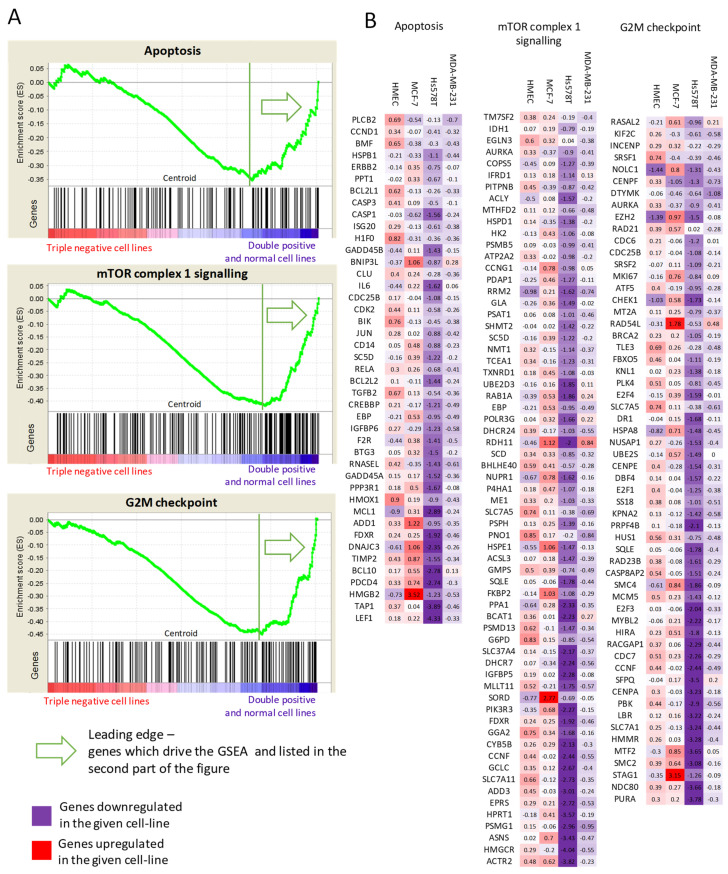
Gene Set Enrichment Analysis. (**A**) Genes in TNBC vs. DPBC and normal cell lines are perturbed differently after arsenate treatment with respect to apoptosis, mTOR signaling and G2M checkpoint signaling (*q* < 0.15). (**B**) Lists of genes differentially expressed per cell line belonging to the above pathways are colored by their differential expression. It can be seen that apoptosis, mTOR signaling and G2M checkpoint regulating genes are downregulated in the TNBC cases after arsenate treatment, while they are upregulated or not changed in the DPBC and in HMEC cell lines. The full results can be seen in Table S4.

We used regularized discriminant analysis (RDA) to differentiate the response to arsenate between the cell lines as follows. For this kind of comparison, we used the fold change values as input. We treated the two TNBC cell lines as one and the normal and DPBC cell line together as a second set of cell lines. This way we intended to investigate whether the RDA analysis will show transcriptomic changes according to the morphological results obtained. Indeed, we found enrichment in the apoptosis, the mTORC and the cell cycle hallmarks using the RDA value as input data (Figure 5). This suggests that even though the hallmarks are not changed at the individual cell line level after arsenate treatment, their response on the transcriptomic level is different when we compare the DPBC and TNBC cells.

The mTORC signalling was differentiated between the TNBC and the DPBC/normal cell lines. The mTORC signalling was generally downregulated in the TNBC cell lines. This downregulation included various metabolic enzymes such as glucose-6-phosphate dehydrogenase (G6PD) or sorbitol dehydrogenase (SORD), amino acid transporters such as cystine/glutamate transporter (SLC7A11) and large neutral amino acid transporter small subunit 1 (SLC7A5). The mTORC is the master regulator of autophagy, inhibiting it in the case of adequate metabolic flux [39]. These results show the downregulation of the metabolic input after arsenate treatment, which can trigger autophagy through the mTORC complex in TNBC cell lines.

However, all these responses on the transcriptome are weak, possibly due to the low concentration of the arsenate treatment used. Next we selected the key regulators of the various processes (autophagy, apoptosis, cell cycle) for further validation experiments to validate our results. The activation of apoptosis, autophagy and cell cycle arrest are the key outcomes of arsenate treatment as observable from the genes shown in Figure 5 in combination with autophagy and apoptosis assays (Figure 1) and microscopic images (Figure 2 and Figure 3).

**Figure 6:**
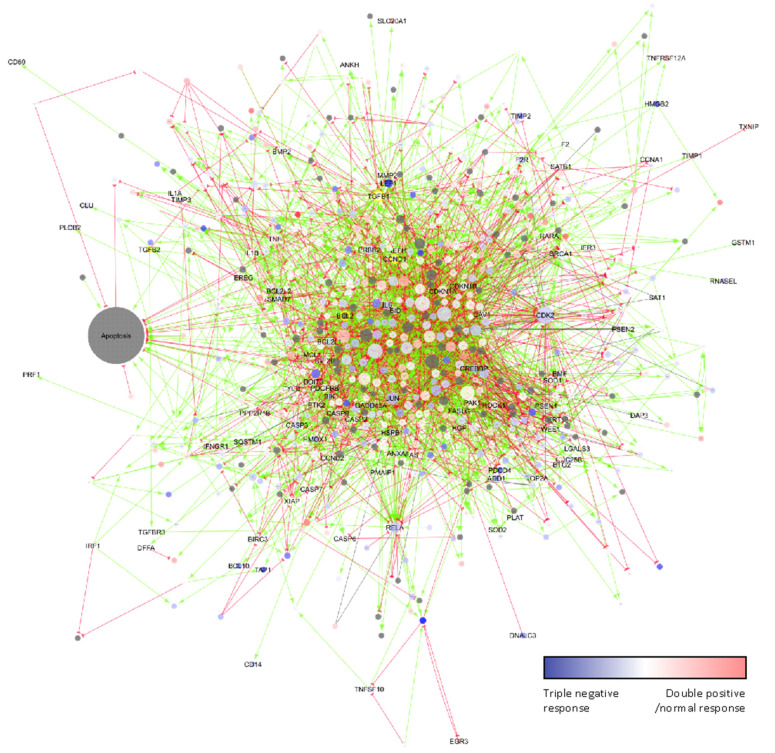
The effect of arsenate treatment on the apoptosis network and its interactors according to the SIGNOR database. Node size is according to degree. The colours are according to centroids above towards the TNBC cell lines. Grey proteins have no centroid values. Green arrows are up-regulating interactions; red half circle-ended lines are downregulating interactions. The effect of grey lines is unknown. High degree yellow bordered proteins are chosen to validate. They are central members of the network in apoptosis. Many high degree proteins such as LEF1 and CDK2 also respond to treatment in TNBC cell lines.

Given that apoptosis regulation is a complex process, we next used network visualization to gain further insights into the mode of action of arsenate treatment (Figure 6). The higher degree proteins tend to be differentially regulated in the TNBC cell lines, such as Lymphoid Enhancer Binding Factor 1 (LEF1) or the cyclin dependent kinase 2 (CDK2). We selected the main regulators of the apoptotic pathway and other proteins which are involved in different processes for further analysis, namely the apoptosis regulator BCL2, the BCL2 domain-containing protein Myeloid Cell Leukemia 1 (MCL1), transforming growth factor 2 (TGFβ2) and Cyclin D1 (CCND1).

The genes were selected based on their function in apoptosis according to the network figure (Figure 6) and their involvement in other biological processes such as cell cycle—CCND1 and TGF pathway TGFB1.

### 2.6. qRT-PCR Validation of Transcriptomic Profiles

**Figure 7:**
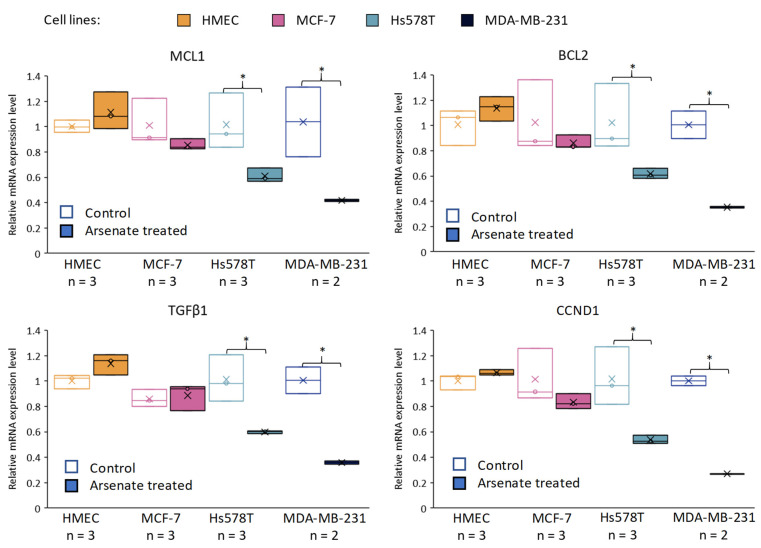
Validation of the effect of arsenate by qRT-PCR on selected genes related to apoptosis and cell proliferation. Relative gene expression levels are shown for MCL1, BCL2, TGFβ1, CCND1 (Cyclin D1) across cell lines and in arsenate treated and control group (untreated cells). The data were normalized to β-actin and B2M using the ΔΔct method for the HMEC, MCF-7, Hs578T and MDA-MB-231 cell lines compared to the arsenate treated group versus the control group. x is the mean and the line is the median in the boxplots (* *p* < 0.05). It can be seen that both apoptosis inhibitors (MCL1 and BCL2) are downregulated after arsenate treatment in TNBC cell lines when compared to untreated cells, but not in the normal and DPBC cell lines. Also, survival factor TGFβ1 and cell proliferation indicator CCND1 are downregulated in Hs578T and MDA-MB-231 cell lines compared to their expression in HMEC and MCF-7.

Following microarray-based gene expression analysis, we selected four genes for qRT-PCR in order to validate our results, which have a central role in apoptosis (Figure 7) and which were negatively regulated from the microarray data as an effect of arsenate treatment.

BCL2 is an apoptosis regulator which blocks BAX from releasing Cytochrome C out of the mitochondria. This represents the initiation step of the intrinsic apoptotic process and activates the caspase cascade [40]. MCL1 has a similar role as a BCL2 family apoptosis inhibitor protein [41], while TGFβ1 is a key cytokine involved in drug-resistance by regulating stemness, epithelial-mesenchymal transition (EMT) angiogenesis, and apoptosis [42,43] The fourth gene, cyclin D1, is one of the cell proliferation cyclins [44] and it has been selected for further analysis because of its prognostic significance in breast cancer patients [45,46].

It can be seen (Figure 7) that in the case of the Hs578T and MDA-MB-231 cell lines, we observed a downregulation of BCL2, MCL-1, TGFβ1 and CCND1 at 24 h post-treatment with arsenate when compared to the control group. No alteration of relative gene expression can be seen in the case of normal cell line HMEC and MCF-7 (Figure 7).

The expression of antiapoptotic regulators (BCL2 and MCL-1) hence significantly decreased after arsenate exposure, which provides a mechanistic rationale for in apoptosis facilitation via the intrinsic apoptosis pathway [47]. CCND1 is an influential cell-cycle regulatory protein, and its overexpression is connected with cell proliferation, poor prognosis and recurrence in breast cancer, which has here shown to be downregulated as an effect of the arsenate exposure. Hence, decreased CCND1 expression can be related to decreased cell proliferation [48]. CCND1 provided to be a link between degradative autophagy and cell cycle regulation in hepatocarcinoma tumorigenesis [49]. Overall, we can see that arsenate regulates key genes involved in cell cycle regulation, signal transduction, autophagy [50] and apoptosis [8,51]. The alteration produced might in involve epigenetic components in addition to the transcriptomic level, [8] which, however, was outside the scope of the current study.

### 2.7. BCL2 Quantification by Fluorescence Confocal Microscopy and TGFβ2 Protein Quantification via ELISA

**Figure 8:**
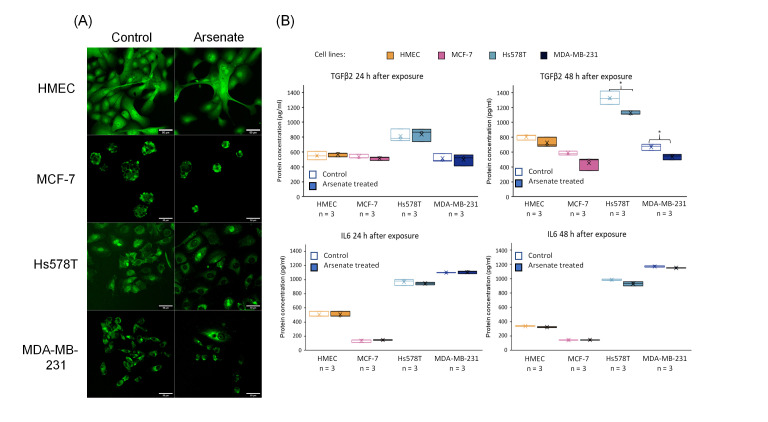
(**A**) Microscopy visualization of BCL2 validation at the protein level. Protein expression of BCL2 marked by fluorescently tagged antibodies followed by confocal microscopy evaluation. It can be seen that the expression level of the BCL2 protein is slightly reduced in case of the arsenate treated group compared to control for the case of Hs578T and MDA-MB-231 at 48-h post-treatment, confirming the qRT-PCR and microarray data. (**B**) TGFβ2 and IL6 validation at the protein level. Protein expression of TGFβ2 and IL6 released in cell culture medium 24 h and 48 h for control and arsenate treated cells (HMEC, MCF-7, Hs578T and MDA-MB-231) evaluated by ELISA. x is the mean and median is the middle line in the boxplots. * *p* < 0.05 two-sided *t*-test. TGFβ2 is downregulated in protein level after 48 h of arsenate treated cells, but there is no change in the IL6 levels.

We next quantified alteration at the protein level as a validation step for the alteration on the transcriptome level. The results from BCL2 protein quantification by confocal immunofluorescence are presented in Figure 8, revealing slightly reduced fluorescence intensity, thereby confirming the reduced expression level of BCL2 as an effect of arsenate exposure (Figure 8A).

Finally, we quantified TGFβ2 and IL6 by an ELISA assay (at the protein level) after 24 h and 48 h from cultures of HMEC, MCF-7, Hs578T and MDA-MB-231 cells for treatment vs. control, the results of which are shown in Figure 8B. We observe a slightly decreased level of TGFβ2 after 48 h in TNBC cell lines. TGFβ2 is involved in the EMT involved in cell migration and angiogenesis, [42] and overexpression of TGFβ2 promotes tumor growth and invasion, therefore its inhibition by arsenate exposure might contribute favorably to treatment efficacy [52]. TGFβ also influences TNBC cancer stem cells through regulating stemness EMT and apoptosis [43]. Downregulating TGFβ2 with arsenate could in turn help to reduce such effects and make the TNBC cells more susceptible to conventional chemotherapy. In contrast to TGFβ2, IL6 had a very little downregulation after 48 h of arsenate treatment in the TNBC cell lines (*p* < 0.05 *t*-test, Figure 8B). IL6 is an activator of mTOR signalling, which is involved in metastasis formation of TNBC [53,54] as well as in drug resistance which is counteracted by the administration of arsenate [33].

## 3. Discussion

Arsenic derivatives showed antitumoral activity in the case of many types of cancer such as arsenic trioxide on head and neck tumors [55] or on epithelial ovarian cancer, Ref. [56] human neuroblastoma, Ref. [57] human liver cancer cells, Ref. [58] leukaemia, Ref. [59] renal cancer [60] and prostate cancer [61]. Arsenate and arsenite showed inhibition of proliferation of melanoma cells [62] and of human promyelocytic leukemia cells [63]; arsenite and arsenic acid induced apoptosis in the leukemia cells [64]; tetraarsenic hexoxide induced G2/M arrest, apoptosis, and autophagy in SW620 human colon cancer cells [65]. In the context of TNBC, arsenic derivatives have shown activity in in vitro experiments against several breast cancer cell lines like arsenate on MCF-7 cells [66]; arsenite on DPBC cells (MCF-7) and TNBC cells (MDA-MB-231, T-47D, BT-20 [7,8,9,10,11], and arsenic disulfide on MCF-7 and MDA-MB-231 breast cancer cells [26,67].

Arsenate derivatives have been researched extensively regarding their medical applicability as well as biological effects. Arsenate affects cancer progression through coding and non-coding genes related to a wide range of biological processes [68]. A particular application of arsenate derivatives is focused on miRNAs as promoters of apoptosis induced by arsenic trioxide, which is commonly used in the treatment of acute promyelocytic leukemia [68,69,70] The cross-talk among all of the literature and the current applicability of arsenate gives a niche for further investigations to fit the puzzle pieces together.

Most of the studies that presented the biological effect of arsenic are related to the oxidation state +3 (arsenite); meanwhile for the oxidation state +5 (arsenate) there is much less information about its known mode of action. Despite the fact that arsenate efficacy in the treatment of breast cancer was demonstrated, Ref. [66] its antitumor mechanism has not been fully elucidated yet.

In a wide range of cellular models [27,71,72,73], it has been shown before that arsenic treatment has the capacity to significantly reduce cell proliferation, invasion, and metastasis and to induce apoptosis. Our analysis now showed that arsenate’s effect is largely cell line specific to TNBC cell lines, absent in HMEC normal control cells, and present only to a much lesser effect in MCF-7 cells. Arsenic treatment has been demonstrated to specifically activate apoptosis in MCF-7 2D- and 3D-culture models [67]; however, arsenate has only a moderate effect on the MCF-7 cell line in the current study. The cause could be that cells were grown in clusters in our study, and darkfield microscopy showed apoptosis only at the edge of the clusters. The effect of arsenate treatment was more pronounced in the case of TNBC cells, as this could be observed by microscopy data and confirmed on the gene expression level.

Arsenite showed the ability to induce S-phase arrest, autophagy and apoptosis on various tumors by modulating genes such as Forkhead box O3 (FOXO3a) and Cyclin D1 (CCND1) [74], or sustaining inhibition of mTORC1 [7], the latter of which was shown to be related to autophagy regulation. The mTOR pathway is activated through IL6 signaling, which is closely related to cell growth and metastasis in TNBC [53,54]. Other studies have shown that the blockade of IL6-associated inflammation positively correlates with the inhibition of tumor growth and EMT process, [53] which should be further explored in TNBC. The mTOR pathway is a frequently activated pathway in human cancers, representing an attractive target for anti-cancer drug development [75]. Furthermore, mTOR also negatively regulates autophagy [76]. The inhibition of mTOR signaling can decrease cellular proliferation and promotion of cell death including apoptosis and autophagy [76]. The current study proposes autophagy and apoptosis as a final cellular response of arsenate-inducing oxidative stress, where mTOR signaling has an essential role, as we observed in our study.

It was demonstrated previously that the apoptotic and autophagic responses have very specific cross-talk [77]. Evidence in the literature suggests that in the case of the TNBC cells, arsenate could induce apoptosis through autophagy. In our experiments, we have seen both elevated autophagy and apoptosis in TNBC cell lines, but not in HMEC cells. MCL1 and BCL2 are the main effector proteins in regulating the antiapoptotic and anti-autophagy response, which were downregulated in TNBC cells validated by qRT-PCR. The apoptosis mechanism was activated in the case of breast cancer cells in this study as well. Similarly, in HT-29 colorectal cancer cells, activation of the intrinsic apoptosis pathway was demonstrated via upregulation of BAX and downregulation of BCL2 [78]. Although this effect was also observed in the present study, it was considerably smaller. Additionally, we have seen the capacities of BCL2 family proteins to regulate autophagy via the interaction with Beclin-1; caspases have been indicated to suppress autophagy via a mechanism mediated by the cleavage of autophagy-related proteins [79].

The analysis described in our paper shows that arsenate reduced cell proliferation as well as activation of autophagy and apoptosis in breast cancer cells. In this study the cytotoxic effect of arsenate was found to be largely cell type specific, as observed previously also in hepatocellular carcinoma cells [80]. Our study next investigated the cellular effects of arsenate further, based on functional tests in combination with transcriptomics experiments to elucidate its mode of action.

**Figure 9:**
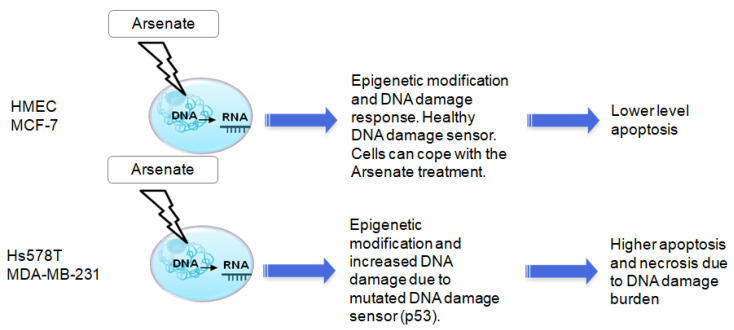
The putative mechanism of action and cell line selectivity of arsenate for HMEC and MCF-7 cells compared to Hs578T and MDA-MB-231; HMEC and MCF-7 cells have intact DNA repair and are hence better able to cope with this type of damage, while TNBC cell lines are known to be TP53 mutant and therefore arsenate causes increased apoptosis and autophagy.

Each cell line in this study responded to arsenate treatment differently (possibly depending on the mutations present, TNBC cell lines being known to be TP53 mutant [81]). In Figure 9 we emphasized the relevance of breast cancer’s molecular subclassification. While arsenate causes increased apoptosis and autophagy in TNBC cell lines, HMEC and MCF-7 cells have intact DNA repair pathways and are therefore better able to cope with this type of damage (Figure 9).

In the case of Hs578T cells, we observed an alteration of chromatin pathways, DNA replication and telomere signaling pathways, and chromatin modification (a form of late apoptosis signals) correlating with microscopy data from previous studies [82,83]. Chromatin modifications are a frequent event observed during the repair of environmental exposure-induced DNA damage, including for arsenic exposure [84]. It has previously been demonstrated that arsenic affects chromatin silencing pathways in HeLa cells [85]. These alterations might be transient or can be accompanied by heritable epigenetic alterations at some specific sites of chronic arsenic exposure. These epigenetic changes and DNA damage might, in turn, be exploited as a therapeutic strategy for breast cancer by inducing apoptosis in TNBC cells. In the case of MDA-MB-231 cells the reduction of cell proliferation was related to the activation of cell death via the endoplasmic reticulum and mitochondrial axis, as confirmed by fluorescence microscopy and gene expression data, whereas in the case of microarray data we identified genes that regulate these processes, which is shown in Figure 6 (network showing the specific genes related to the intrinsic, mitochondrial axis of apoptosis via MCL1 and BCL2) [86]. Arsenic compounds activate an apoptosis-related mechanism via intrinsic and extrinsic caspase pathway activation [87].

Arsenate target genes involved epigenetic reprogramming [88], which lately affect cell fate through a direct or indirect way [8]. DNA damage caused by arsenic derivatives exposure was identified in multiple cancer models and was demonstrated to affect the response to chemotherapy [89]. In the current study, arsenate has shown a chromatin-modifying effect in all cell lines, which can be the marker of DNA damage, but the normal and DPBC cell lines can more readily cope with this effect, which resulted in a relatively decreased apoptotic rate compared to the TNBC cell lines with relatively higher apoptotic levels, leading to a degree of selective toxicity [82,83]. The mutated status of p53 in the two TNBC cell lines is possibly causally related behind the decreased DNA damage response [22]. In spite of the fact that we treated the cells with a very low concentration of arsenate at 50 nM, it had the capacity to interfere with cell proliferation checkpoints and apoptosis and thus suppress tumorigenesis. This has important relevance because DNA repair systems interact with other cellular components responsible for homeostasis and DNA metabolism [90]. Arsenic is presented in the literature not only as an apoptosis regulator but also as an autophagy regulator, which in agreement with our data, and also for the +5 oxidation state.

Our data suggest the involvement of apoptosis and autophagy in the effects of arsenate exposure, which furthermore appears to be specific to cancer cell lines, based on the data generated here. In our in vitro experiments we have not distinguished whether arsenate or arsenite had the biological effect in the intracellular milieu. Nevertheless, the outcome of the apoptosis and autophagy assay suggest a cell line specific cytotoxic effect.

Arsenic in oxidation state +3 has previously been found to interfere with intrinsic and extrinsic apoptosis and autophagy mechanisms in the breast cancer cell lines MCF-7 and MDA-MB-231 [26]. Arsenic trioxide was found to suppress cell growth, to stimulate apoptosis, and to be involved in retarded cell invasion by interfering [91] with coding and non-coding gene regulation [27,28,29,30]. We can conclude that in our case, the arsenate exposure activates two important mechanisms, autophagy and apoptosis regulating the cell death, and hence we consider arsenate as a promising candidate in cancer management.

## 4. Materials and Methods

### 4.1. Cell Lines and Treatment

HMEC (human mammary epithelial cells, A10565 Life Technology, Carlsbad, CA, USA) were maintained in HMEC basal serum free medium (Life Technology, cat no. 12753018, Carlsbad, CA, USA) and HMEC supplement kit (Life Technology cat. No. 12755013, Carlsbad, CA, USA). The DPBC cell line MCF-7 (ATCC collection, Manassas, VA, USA) was cultured in MEM medium supplemented with 10% fetal bovine serum, 2 mM L-glutamine and 1% nonessential amino acids. The Hs578T cell line (ATCC collection) was maintained in MEM (Dulbecco’s Modified Eagle Medium, Gibco Life Technologies, Waltham, MA, USA) high glucose (4500 mg/mL glucose) supplemented with 10% fetal bovine serum, 2 mM L-glutamine, 1% nonessential amino acids (Gibco Life Technologies, Waltham, MA, USA) and 0.01 mg/mL insulin. The MDA-MB-231 cell line (ATCC collection) was cultured in RPMI-1640 medium supplemented with 10% fetal bovine serum and 2 mM L-glutamine. All cells were maintained in a humidified incubator at 37 °C with 5% CO_2_.

We used the As^5+^ in solution directly because arsenate is internalized easily by phosphate carriers. [92] Hence, the normal and tumor breast cancer cells were treated with arsenic in the oxidation state +5 (As^5+^) presenting less direct cell toxicity than (As^3+^). [14] Arsenate was obtained in 1000 mg/L standard solution (As + 5HNO_3_ → H_3_AsO_4_ + 5NO_2_ + H_2_O) (produced by Merck KGaA, Darmstadt, Germany, Product Number 1197730100, Lot number HC55536773) and diluted to the required concentration.

### 4.2. Colony Assay

Treated and untreated cells were seeded in six-well plates at a density of 250 cells/well/2 mL in triplicate. After 14 days, the cells were washed with PBS 1×, fixed with 1 mL of methanol 80% for 15 min, stained with 300 µL of Trypan Blue 0.2% and then washed with PBS 1×. The colonies were counted by a visual observer without the use of visual augmentation devices. Images of the plates have been taken with a c300 machine (Azure Biosystems, Dublin, CA, USA) using the white light and then they were counted directly from the plate (*n* = 3). A graphical representation and *t*-test results were shown.

### 4.3. Autophagy and Apoptosis Detection

Both autophagy and apoptosis assessments were done using fluorescence microscopy on 10,000 pre-plated cells for 24 h in 96-well plates for each triplicate of control and arsenate treated samples. Fluorescence microscopy was performed on an Olympus I×71 microscope (Olympus, Tokyo, Japan) using a 20× objective for magnification.

For autophagy detection, the cells were treated with an Autophagy/Cytotoxicity Dual Staining Kit (Abcam cat no. ab133075, Cambridge, MA, USA) that contains monodansylcadaverine (MDC) for autophagic vacuole detection in cultured cells and propidium iodide (PI) for necrotic cell detection. Staining was applied after 24 h of arsenate treatment. Apoptosis was detected by the Annexin V-FITC/PI Apoptosis Detection Kit (Abcam cat no. ab14155, Cambridge, MA, USA). The kit contains Annexin V-FITC that stains in green the apoptotic cells that translocated membrane phospholipid phosphatidylserine to the outer leaflet of the cellular membrane, while the PI part of the composition stains the nuclei. Late apoptotic cells are hence double stained with both PI and Annexin-V-FITC. The staining was performed according to the manufacturer’s protocol followed by fluorescence microscopy evaluation (20× magnification). On four different images the apoptotic and autophagic cells were counted in both, treated and untreated conditions. The average cell count of the untreated condition was set to 100% and changes in cell number were reported as multiples of this number.

### 4.4. Dark-Field Microscopy

Dark-field microscopy was performed using an Olympus B × 43 microscope (Olympus, Tokyo, Japan) equipped with a CytoViva Enhanced Dark-Field Condenser (Cytoviva, Auburn, AL, USA), an UPlanFLN60×, NA = 1.2 oil immersion objective (Olympus, Tokyo, Japan) and a 6.4 µm/pixel CCD camera (QImaging, Surrey, BC, Canada). Images were calibrated for scale and annotated in ImageJ2.0 [93] and converted to 8-bit grayscale. Contrast enhancement was performed in the same software (0.3% saturated pixels; Normalized and Histogram equalized) followed by the application of an Unsharp Mask (Sigma value = 2–12 pixels; Mask Weight = 0.6). The magnification used for all images was 60×.

### 4.5. Cytoskeletal Evaluation

The fluorescent staining protocol used DAPI (Abcam, Cambridge, UK) for the labelling of the nucleus and Phalloidin-FITC (Cytoskeleton Inc., Denver, CO, USA) for the cytoskeleton. After treatment, the cells were fixed in 4% paraformaldehyde followed by 0.5% Triton × permeabilization for 1 h. The cells were incubated at 37 °C and 5% CO_2._ Thereafter, 100 µL of 200 nM Phalloidin-FITC was added and the samples were incubated at room temperature under no illumination for 30 min. 200 µL of 100 nM DAPI was added over the coverslip for 30 s and washed with a phosphate saline buffer. The coverslips were mounted with 90% glycerol. Images were captured using a UPLSAPO40 × 2, NA:0.95 objective (Olympus, Tokyo, Japan) and excitation wavelengths/emission windows were automatically selected according to the fluorescence dye spectral information database inside the acquisition software (FW10-ASW, Olympus, Tokyo, Japan).

### 4.6. Microarrays

For microarray experiments, cells from three serial passages were seeded in a six-well plate, using 0.3 million cells/well for each triplicate of control and arsenate treated cells. RNA extraction was performed using TriReagent, which was then purified using the RNeasy Mini kit (Qiagen, Hilden, Germany).

The microarray samples were prepared according to the Agilent Low Input Quick Amp Labeling (5190–2305) protocol to synthesize equal quantities of 100 ng of total RNA, followed by purification of the hybridization products using the RNeasy Mini kit (Qiagen). A NanoDrop2000 spectrophotometer (Thermo Scientific, Waltham, MA, USA) was used to perform probe quality control, with results showing that all the probes had a specific activity higher than 6 pmol/µL Cy3/µg cRNA (specific activity > 8 pmol Cy3/µg cRNA). Fragmentation and hybridization were performed based on the Agilent One-Color protocol (Agilent Technologies, Santa Clara, CA, USA). [94,95] The samples were hybridized for 17 h at 65 °C in a hybridization oven. This was followed by microarray slide scanning with a SureScan Microarray Scanner (1 × 60 k array slides with 61 × 21 mm size, resolution 3 µM) from Agilent, and image processing was undertaken with the Feature Extraction 11.0.1.1 software (Agilent 2016, Santa Clara, CA, USA).

Gene expression values were determined using the Agilent G4851C microarray slides for the three arsenate treated samples and three controls across four cell lines (HMEC, MCF-7, Hs578T, and MDA-MB-231).

Resulting data were analyzed using the limma [96] package in R [97], where the background was corrected with the “*normexp*” method and then quantile normalization [96] was performed, as we used in our previous work. [98] Probes transcribed and expressed in at least three samples in any conditions at a level higher than the 95th percentile were selected. This process resulted in a list of 29,874 probes. Next, probes were mapped to genes using the mean expression with the “*avereps*” function in R. The probe sets were translated using the annotation file of the microarray chip [97]. This resulted in a list of 18,849 genes which were used for subsequent analysis, following the standard procedure [96]. From the gene expression values, we conducted a Principal Component Analysis (PCA) using the “*prcomp*” function in R [97] for visualization purposes.

Based on the experimentally determined gene expression profiles, we calculated the average log2 fold change value per gene for each cell line responding to arsenate in R, using the functions lmFit and eBayes [99]. Significantly differentially expressed genes (with Benjamini-Hochberg corrected *p*-value < 0.1 and |log2 FC| > 1) were tested in the Gorilla [100] tool for Gene Ontology Biological Process overrepresentation [101,102]. For gene set enrichment analysis we used the CAMERA method [103].

The calculation of log2 fold changes between each treatment and each non-treated sample resulted in nine fold-change values per cell line (three treated and three untreated replicates in each distinct cell lines). Next, Robust Regularized Discriminant Analysis (RDA) was performed on these fold changes using the R package RDA [104]. After a parameter search we chose to use as parameters α = 0.22 and δ = 0.33, because these values correctly classified all of our samples (see Table S1 for the confusion matrix).

After optimizing parameters, we calculated a centroid value per gene, which indicates the extent to which the given gene is able to differentiate the two TNBC cell lines from the double positive and normal cell line. This centroid was the subject of the subsequent Gene Set Enrichment Analysis (GSEA) [105] using the Cellular Hallmarks from MolSigDB and the network analysis (see below) [106]. A high centroid-based differential expression value represents a larger response of the given gene in the TNBC cell lines. The cut-off for significantly differentially regulated hallmarks was set to an FDR of below 0.15. The GSEA was run using a gene set size cut-off larger than 10 genes, but smaller than 500. All other parameters were kept as default.

### 4.7. Apoptosis Network in Pathological Condition as Effect of Arsenate Treatment

We next generated an apoptosis reference network by using the genes from the MolSigDB apoptosis pathway [106] and mapped them to UniProt gene identifiers through the UniProt mapping service [107]. We used the mapped UniProt identifiers as a searching seed in the SIGNOR database [108]. We kept the seeds and also their direct interaction partners if they interacted with at least two seed proteins. We then mapped the centroid values of each gene from the RDA analysis to the network, and we indicated that with a gradient. We calculated the degree—number of neighbours—of all nodes of the whole SIGNOR network. Degree was indicated by node size in the visualization. This method visualizes the most central regulators in the apoptosis specific regulatory network as a key anticancer mechanism.

### 4.8. qRT-PCR Evaluation

Total RNA extraction was performed using TriReagent (Invitrogen, Carlsbad, CA, USA) according to the manufacturer’s protocol. A NanoDrop-1100 (Thermo Fisher Scientific, Carlsbad, CA, USA) was used to evaluate RNA concentration and quality by measuring the absorbance of UV light. For gene expression evaluation, total RNA (1000 ng) was reversely transcribed into cDNA using the High Capacity cDNA Reverse Transcription Kit (Applied Biosystems, Carlsbad, CA, USA). We used the Assay Design Center from Roche for the primer design (Roche Inc. 2018, NJ, USA). The primers for each gene are listed in Table S2. SYBR Select Master Mix (Life Technologies, Carlsbad, CA, USA) was used for gene expression evaluation, and all amplifications and detections were carried out in the Applied Biosystems ViiA7 System (Thermo Fisher Scientific, Waltham, MA, USA) based on the manufacturers recommended protocol.

### 4.9. TGFβ2 and IL6 Quantification in Cell Culture Medium

The expression level of TGFβ2 released in the cell culture medium was detected by ELISA using the Human TGF-beta 2 DuoSet ELISA (R&D System, cat no. DY302, Minneapolis, MN, USA), DuoSet Ancillary Reagent Kit 2 (5 plates, R&D Systems, cat no. DY008, Minneapolis, MN, USA) and Sample Activation Kit 1 (R&D Systems, cat no. DY010, Minneapolis, MN, USA). For IL6 quantification from cell culture, ELISA was performed using the IL6 DuoSet ELISA Kit (R&D System, cat no. DY206-05, Minneapolis, MN, USA) along with the DuoSet Ancillary Reagent Kit 2 (5 plates, R&D Systems, cat no. DY008, Minneapolis, MN, USA).

### 4.10. BCL2 Protein Evaluation by Confocal Microscopy

For immunofluorescence staining, a Human/Mouse BCL2 Antibody (R&D Systems, cat no. AF810-SP, Minneapolis, MN, USA) was used. Incubation was done with 5 µg/mL overnight followed by washing steps and incubation for 2 h with secondary antibody Goat Anti-Rabbit Alexa Fluor^®^ 488 (ab150077, 1:100 dilution). Laser scanning confocal microscopy was performed on an Olympus FV1200MPE microscope equipped with UPLSAPO40×2, NA:0.95 objective.

### 4.11. Statistical Evaluation

For pairwise comparisons we used two-sided *t*-tests, our considered significance level was *p* < 0.05, and the statistical analyses were performed using GraphPad Prism software version 6 for Windows (GraphPad Software, San Diego, CA, USA). For microarray differential gene analysis, we used moderate t-statistics with a Benjamini-Hochberg correction and *p* < 0.05. For Gene-set enrichment analysis we used the Kolmogorov Smirnov test for *p* < 0.05 and FDR < 0.1.

## 5. Conclusions

In this study we were able to demonstrate that arsenate induces a cell line specific morphological and transcriptomic alteration at low concentration. Arsenate induces the cytoskeletal alteration and cell death in TNBC cell lines through activating autophagy and apoptosis and reduces the clonogenic capacity.

The novelty of this study stands in the fact that arsenate therapeutic stress produces pro-apoptosis signals largely selectively in TNBC cells (based on dark-field microscopic and cytoskeletal evaluation). In addition, arsenate showed no effects in HMEC cells and only moderate effects in MCF-7 cells.

Regularized discriminant analysis showed that the low concentration of arsenate affected the G2M checkpoint, autophagy and apoptosis cell line specifically. The downregulation of anti-apoptotic genes (MCL1, BCL2, TGFβ1 and CCND1) was confirmed by qRT-PCR, and on the protein level, for TGFβ2, by ELISA, concluding that TNBC and non-TNBC cell lines in this particular experimental setup reacted differently to treatment.

The alteration of gene expression levels demonstrates a crosstalk among autophagy, cell cycle and apoptosis as a potential mechanism of action of arsenate which must be investigated in future pharmacological interventions.

This study is a step toward understanding arsenate TNBC-type specific effects which potentially correlates with active DNA repair pathways. However, further studies are necessitated to demonstrate arsenate metabolism and mechanism of action, considering the importance of intracellular reduction of the metalloids for biological effects. Nevertheless, this study makes the use of arsenate to be a potential selective chemotherapeutic drug treating triple negative breast cancer one step closer to reality.

## Data Availability

The data generated or analyzed during this study are included in this published article (and its Supplementary Information files).

## Supplementary Materials

The following supporting information can be downloaded at: https://www.mdpi.com/article/10.3390/ijms23094784/s1.
